# Mental Condition Monitoring Based on Multimodality Biometry

**DOI:** 10.3389/fpubh.2020.479431

**Published:** 2020-10-22

**Authors:** Masashi Kiguchi, Stephanie Sutoko, Hirokazu Atsumori, Ayako Nishimura, Akiko Obata, Tsukasa Funane, Hiromitsu Nakagawa, Masashi Egi, Hiroyuki Kuriyama

**Affiliations:** ^1^Center for Exploratory Research, Hitachi, Ltd., Kokubunji, Japan; ^2^Center for Technology Innovation, Hitachi, Ltd., Kokubunji, Japan; ^3^Global Center for Social Innovation, Hitachi, Ltd., Kokubunji, Japan

**Keywords:** multimodality, multivariate linear regression, PC logger, activity tracker, near-infrared spectroscopy (fNIRS)

## Abstract

We have developed a system with multimodality that monitors objective biomarkers for screening the mental distress in the office. A field study using a prototype of the system was performed over four months with 39 volunteers. We obtained PC operation patterns using a PC logger, sleeping time and activity levels using a wrist-band-type activity tracker, and brain activity and behavior data during a working memory task using optical topography. We also administered two standard questionnaires: the Brief Job Stress Questionnaire (BJS) and the Kessler 6 scale (K6). Supervised machine learning and cross validation were performed. The objective variables were mental scores obtained from the questionnaires and the explanatory variables were the biomarkers obtained from the modalities. Multiple linear regression models for mental scores were comprehensively searched and the optimum models were selected from 2,619,785 candidates. Each mental score estimated with each optimum model was well correlated with each mental score obtained with the questionnaire (correlation coefficient = 0.6–0.8) within a 24% of estimation error. Mental scores obtained by means of questionnaires have been in general use in mental health care for a while, so our multimodality system is potentially useful for mental healthcare due to the quantitative agreement on the mental scores estimated with biomarkers and the mental scores obtained with questionnaires.

## Introduction

Mental disorders are of significant concern in terms of not only public health but also economic development and social welfare (1). Depression affects over 120 million people and causes long absences from work and increased risk of suicide. Organization for Economic Co-operation and Development (OECD) reported that mild-to-moderate mental disorders affect around 20% of the working-age population in the average OECD country and predominantly include highly treatable disorders such as anxiety and depression. Although treating depression in primary care is feasible and very cost-effective, studies have shown that 56.3% of patients do not get sufficient care (2). Furthermore, U.S. workers suffering from depression cost employers an additional 31 billion dollars each year in lost productive time (3). A key management issue facing enterprises today is ensuring that they prevent depression, support the return to work, and prevent recurrence. Many companies have services for managing employees' mental condition, such as the employee assistance program (EAP)^1^. Because the mental distress is a risk factor of depression, early detection of risk factors could be exploited for prevention purposes. Therefore, the accurate and low-cost monitoring of mental condition is required for screening distress in office.

Information and Communication Technology (ICT) and Internet of Things (IoT) have the potential to provide a low-cost condition monitoring system with high accuracy for mental healthcare. Especially, the mental distress levels observed using questionnaires are widely used for screening. A variety of devices for monitoring mental stress have already been developed (4). We previously developed a PC logger, a wrist-band type activity tracker, and a wearable optical topography as a non-invasive brain activity sensor. These have been independently used in several studies on mental healthcare application. The fractal dimensions obtained from the PC logger data are expected to be related with mood states (5). The usage of the wrist-band type activity tracker for judgments of reinstatement reduced the ratio of re-leave (6). The brain activity measured by optical topography during working memory tasks was affected by mood state (7–9). The optical topography measurement was applied to return-to-work trainees in remission of mental disorders with depressive symptoms (10). In this paper, we introduce a multimodality data acquisition system we developed to combine these devices for monitoring multilateral conditions of mental health. The combination of behavioral and brain measurements is a novel approach.

## Method

### Multimodality Measurements

**Figure 1:**
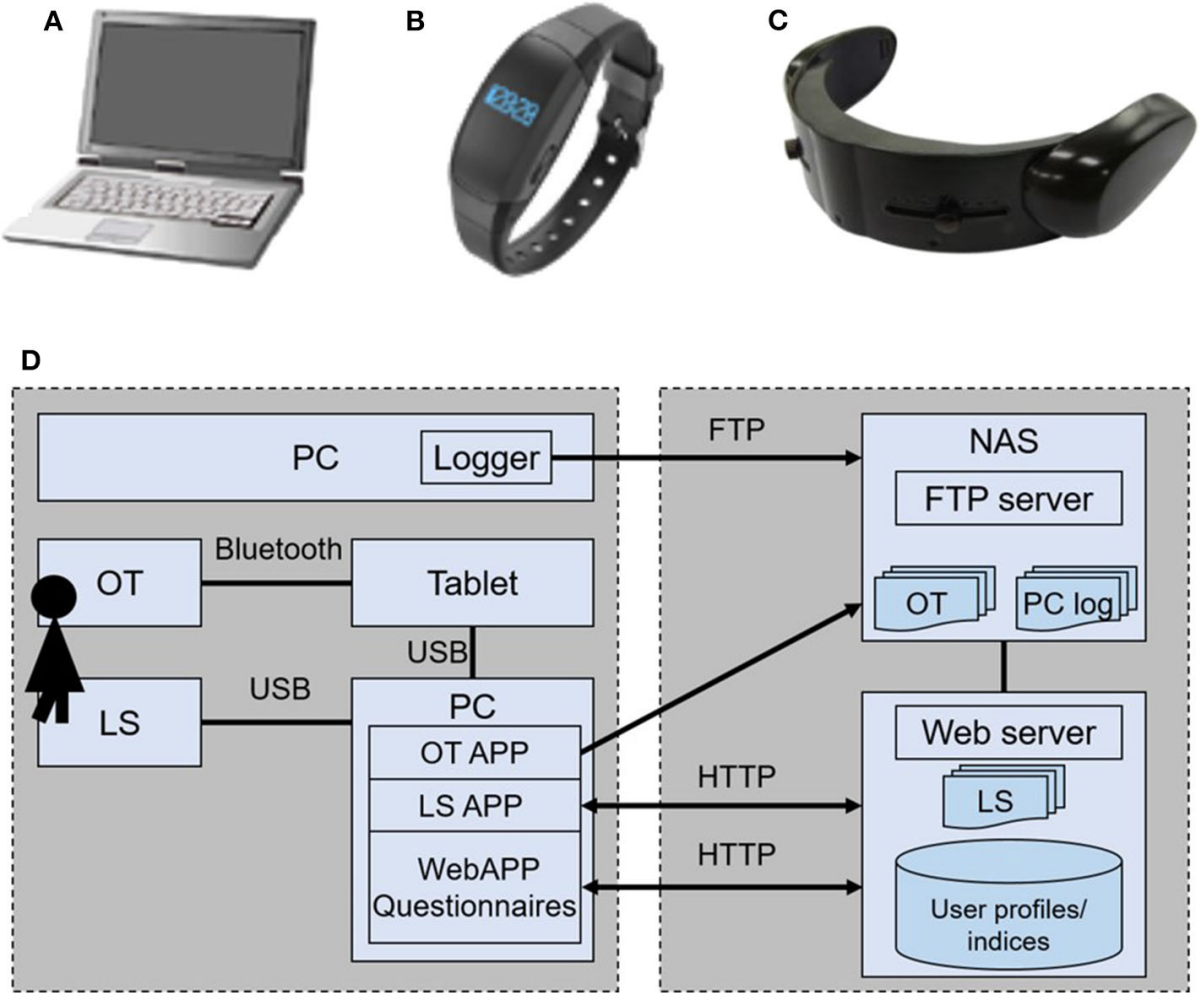
Devices used for monitoring mental conditons: **(A)** PC logger, **(B)** Life Scope (LS), and **(C)** HOT-1000 optical topography (OT). **(D)** The data acquisition and database system.

The equipment and data acquisition system used in this study are shown in Figure 1. The PC logger is our original software developed for the Windows OS. It was installed on each participant's PC and hooked key- and mouse-events with time stamps as a background process. In order to avoid an information security risk, the kind of tapped key, i.e., alphabet key, number key, or special key, was recorded instead of key characters. Mouse button click and mouse movement distance were recorded as well. A cumulative distribution of the key/mouse events frequently showed power of event intervals. These power exponents are related to the total number of event and operator mood states. Using the key/mouse data recorded over the course of a day, a fractal dimension was obtained as a slope of fitted line for cumulative distribution vs. time-interval graph. Because the fractal dimension also depends on the workload in a day, the key/mouse index *d*_*key*/*mouse*_ for the day was obtained as a deviation from the fractal dimension stemming from workload (5).

The wrist-band type activity tracker, Life Scope (LS, Hitachi, Ltd.), is equipped with a triaxial accelerator. All raw signals of the accelerator are stored in its own memory. A variety of features were calculated using the raw data in the server (11). Among them, we used steps, activity strength in metabolic equivalents (METs), and sleeping hours for the analysis.

**Figure 2:**
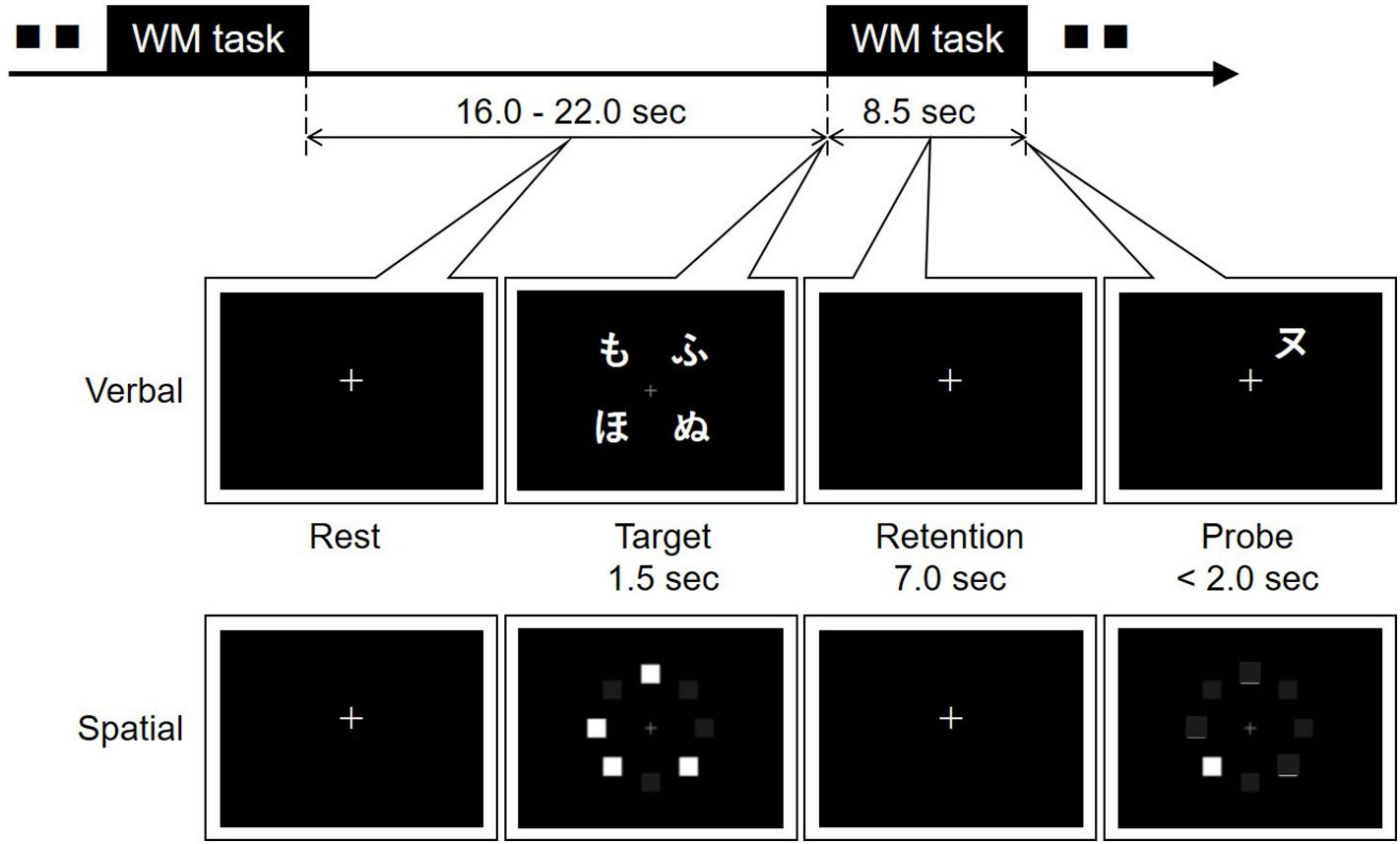
Verbal and spatial delayed matching tasks for optical topography measurement. Four Japanese characters (*hiragana*) or four squares were displayed for 1.5 s on a tablet PC as a target in the verbal or spatial working memory task, respectively. After memory retention for 7.0 s, another Japanese character (*katakana*) or one square was shown as a probe. In the verbal task, participants were asked to click the circle button or the cross button displayed on the tablet PC when the probe character had the same pronunciation as one of the target characters or when it did not. In the spatial task, the agreement of the position between the probe and one of the targets was answered in the same way.

Optical topography (OT) is a tool for measuring cerebral blood volume change associated with brain activity on the basis of Near-infrared spectroscopy (NIRS). Two regions on the forehead were covered by a handy and wireless model, HOT-1000 (Hitachi High-technologies Corporation). The headset was connected through Bluetooth with a tablet PC that provided a task for activating brain function, acquired data, and displayed the results. The brain activity during a working memory (WM) task (namely, a delayed matching task; see Figure 2), was observed by OT for estimating mood states (10). Increases of oxygenated hemoglobin during spatial and verbal working memory tasks at the left and right sides of the forehead were measured. The response time to answer and the rate of correct answers were also recorded.

Each item of time-series data obtained by the PC logger, wrist-band type activity tracker, and optical topography was stored in the time-series analysis database through the local network. The query object graph analysis method was used to enable analysis sharing among analysts by preventing duplicate processing and data explosion (12).

### Questionnaires

The Kessler psychological distress scale (K6) (13) and a part of the Brief Job Stress Questionnaire (BJS) (14)^2^ were used for obtaining mental scores. The K6 score is the standard questionnaire for screening the mental distress. When the K6 score is lower, the condition is better. The cutoff is 5. BJS consists of 52 questions concerning job, health, and people surrounding the respondent. As the situations concerning job and people did not frequently change during the field test, 29 questions concerning health were used for determining subjective mental scores of lassitude, irritation, fatigue, anxiety, depression, and physical stress. All mental scores of BJS were converted into values from 1 to 5 in accordance with the manual^3^. When the mental score is higher, the condition is better. A total value of mental scores lower than 12 is regarded as stressful.

### Trial in Office

Thirty-nine healthy volunteers (32 males, 7 females, 43.7 ± 8.9 years old) with no history of mental disorders participated in the measurement over four months. Neither measured data nor information were fed back to participants so as to avoid any effects on the trial. The data from the volunteers were obtained according to the regulations set forth by the internal review board at the Central Research Laboratory, Hitachi, Ltd., following receipt of their written informed consent.

The PC loggers were installed on all PCs used by participants in the office. The keyboard and mouse operations were recorded the entire time PCs were running. All logs were combined for each participant according to the timestamp. The key index and the mouse index were calculated using data from one day and averaged over one week.

The participants were asked to wear the LS wristband all day except when bathing. Data from the LS was sent to the server through a local area network once a week. The values of steps, METs, and sleeping hours were obtained as averages of one week.

Once a week at the same time, the OT measurements were performed and the questionnaires were administered. While the dates and times for each participant were set in principle, they were nevertheless flexible in order to ensure that enough data were gathered.

### Analysis

A linear multiple regression model was obtained for each mental score using multimodality indices, as

(1) $$\begin{array}{r} y\left[i\right]=c_{0}+\sum_{j}^{m}c_{j}x_{j}\left[i\right] \end{array}$$

(2) $$\begin{array}{r} \varepsilon^{2}=\frac{\sum_{i=1}^{N}{\left(y_{o}\left[i\right]-y\left[i\right]\right)}^{2}}{N}, \end{array}$$

with target variable *y*_*o*_, explanatory variable *x*_*j*_, regression coefficients *c*_*j*_, square error ε^2^, the number of explanatory variables *m*, and the number of data *N*. Target variables were scores of lassitude, irritation, fatigue, anxiety, depression, physical stress, total, and K6. The obtained multimodality indices are listed in Table 1. In order to check the possibility of prediction, each index was observed one week and two weeks before the score sheet was tallied. In total, 51 explanatory variables were obtained.

**Table 1:** The number of explanatory variable and model for each target variable.

**Target variable**	**Explanatoy variable**	**Model**
Lassitude	24	28230
Irritation	31	108551
Fatigue	27	56103
Anxiety	19	6239
Depression	16	2288
Physical stress	20	10354
Total score	16	2816
K6	15	2777
Total	–	217358
Average	21	27169.75

Here, we investigated three cases of *m* = 3, 4, and 5. The total combinations of choosing 3, 4, and 5 indices out of 51 indices equal to 2,619,785 for one mental score. More than twenty million cases of calculation were required for eight mental scores. In order to reduce the number of calculations, each explanatory variable that had a correlation coefficient with each target variable of <0.1 was rejected as a target variable. Also, combinations of the same index for a different week were not permitted. As a result, the number of combinations was reduced to one-hundredth.

The number of the explanatory variables may be different across participants because the measurement schedules did not match the participants' ones (e.g., business trips, days off). When the temporally shifted explanatory variables (i.e., one, two weeks in prior to the measured target variables) were used in regression models, the temporal data of the target variables consequently decreased. Therefore, missing some temporal data inevitably occurred; only data having the complete temporal information were included in the regression analysis. Neither data normalization nor further elimination were performed because there was no observed improvement (data not shown).

The three-fold cross validation was performed to select each optimum model for each target variable. The fold number was determined to ensure that a sufficient amount of data was included in each subset. The subsets were created based on the participant-wise approach to validate participant dependency on models. No data measured from a participant at different measurement times were assigned in different subsets; models with the minimum participant-dependent effect were selected. In order to avoid dropping the models with a larger correlation coefficient *r* and slightly larger ε^2^ than the model with minimum ε^2^, we define an evaluation index *V* for selecting candidates of the optimum model as follows:

(3) $$\begin{array}{r} V=\frac{r-<r>}{\sigma_{r}}-\frac{\varepsilon^{2}-<\varepsilon^{2}>}{\sigma_{\varepsilon^{2}}}, \end{array}$$

where < *r*>, < ε^2^>, σ_*r*_, and $\sigma_{\varepsilon^{2}}$ are mean values and standard deviations of *r* and ε^2^, respectively. Both *r* and ε^2^ were standardized to equally contribute to *V*. The optimum model for each mental score whose ε^2^ was the smallest among ten candidate models identified using *V* was chosen.

## Results and Discussion

Table 1 shows the valid target variables and the numbers of models for each target variable obtained after the reduction described above. The numbers of significant explanatory variables (*x*_*sig*_) and regression models $\left(C_{x_{sig}}^{3}+C_{x_{sig}}^{4}+C_{x_{sig}}^{5}\right)$ were varied across the target variables. The total number of models was 217,358, which means we were able to reduce the computation cost by 1/1000 compared to the initial one.

The explanatory variables are shown in Table 2. The postfix, “_n” means that the explanatory variable was obtained n weeks before the measurement of target variables. For example, Ped_*2* is the mean value of steps in a week obtained two weeks in prior to the measurement of mental scores. The number of data (*N*; the complete temporal data) ranged from about 30 to 70.

**Table 2:** Explanatory variables.

**Name**	**Modality**	**Description**
Ped_*n*	LS	Steps in a day
mets_*n*	LS	Metabolic equivalents in a day
sleep_*n*	LS	Sleeping time in a day
keylog_*n*	BM1	Fractal dimension of key operation in a day
mlog_*n*	BM1	Fractal dimension of mouse operation in a day
ot_s_l_*n*	OT	Left PFC activity during spatial working memory task
ot_s_r_*n*	OT	Right PFC activity during spatial working memory task
ot_v_l_*n*	OT	Left PFC activity during verbal working memory task
ot_v_r_*n*	OT	Right PFC activity during verbal working memory task
ot_sv_l_*n*	OT	Left PFC activity (spatial—verbal)
ot_sv_r_*n*	OT	Right PFC activity (spatial–verbal)
ot_s_rl_*n*	OT	Laterality of PFC activity during spatial working memory task
ot_v_rl_*n*	OT	Laterality of PFC activity during verbal working memory task
ot_s_cr_*n*	OT	Correction rate of spatial working memory task
ot_v_cr_*n*	OT	Correction rate of verbal working memory task
ot_s_rt_*n*	OT	Response time for spatial working memory task
ot_v_rt_*n*	OT	Response time for verbal working memory task

“*_n” indicates that the values were obtained n weeks before the mental score*.

**Figure 3:**
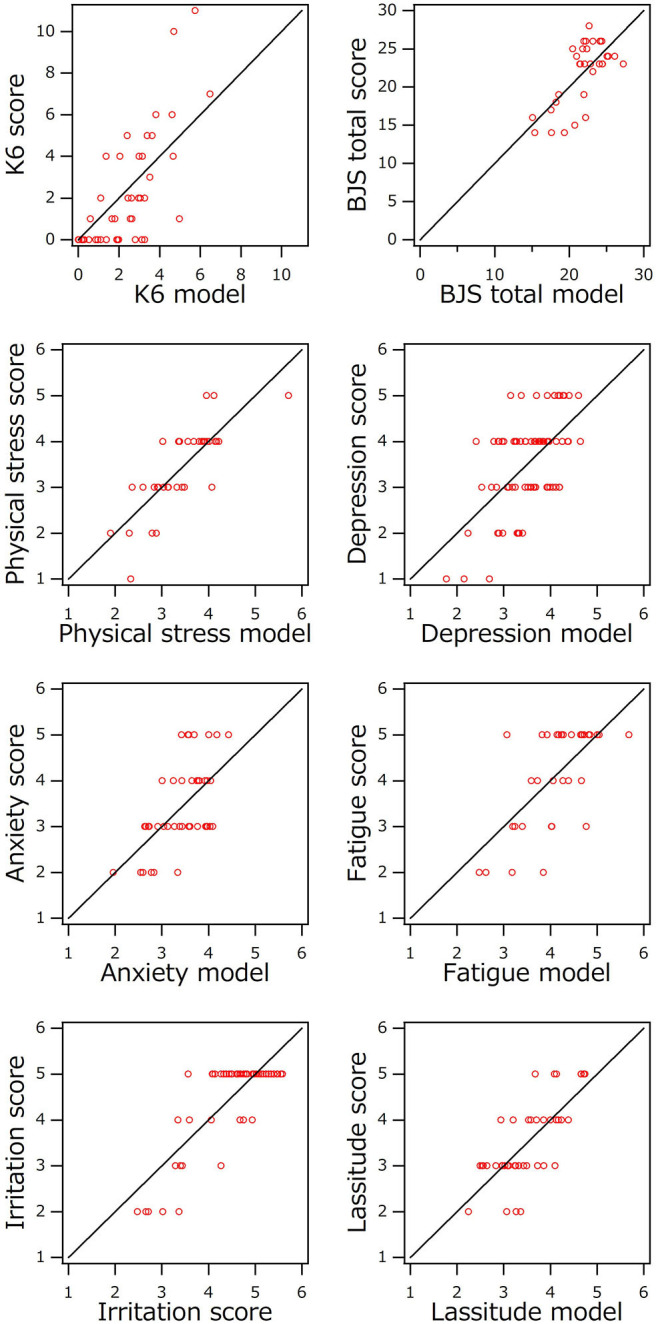
Comparison of mental scores and values estimated using optimized multivariate linear regression model.

Figure 3 shows the relationship between mental scores and values estimated using each optimum model shown in Table 3. The best combination of explanatory variables was selected for each target variable. For example, the best model for depression is described as below.

(4) $$\begin{array}{r} \text{Depression}=1.25+6.53^{*}\text{keylog\_}0+4.06^{*}\text{mlog\_}2\\ \;+2.43^{*}\text{ot\_cr\_}2+0.000149^{*}\text{ot\_s\_rt\_}2. \end{array}$$

No explanatory variables taken one week before the measurement of depression score was used in the above model.

**Table 3:** Optimum model for each mental score with correlation coefficient *r*, estimation error ε, and percentage of ε to full score.

**Mental score**	**c0**	**c1/var**	**c2/var**	**c3/var**	**c4/var**	**c5/var**	***r***	**ε**	***ε [%]***
Lassitude	4.17	−0.00341	0.00316	−0.0257	0.204	−7.82	0.765	0.935	18.7
		ot_s_rt_0	ot_v_rt_0	sleep_1	ot_s_r_1	mlog_2			
Irritation	7.76	0.305	0.00184	−4.36	−2.21	−0.192	0.798	0.780	15.6
		ot_v_l_1	ot_v_rt_1	mets_2	mlog_2	ot_sv_r_2			
Fatigue	3.93	0.352	0.177	−0.0483	−0.000195	−0.233	0.688	1.19	23.8
		ot_v_rt_0	ot_s_r_1	ot_s_rt_1	sleep_2	mlog_2			
Anxiety	−1.65	−0.598	3.53	−0.00108	0.00290	−0.0554	0.624	0.961	19.2
		mlog_0	ot_v_cr_0	ot_s_rt_1	ot_v_rt_1	sleep_2			
Depression	1.25	6.53	4.06	2.43	0.000149		0.627	1.09	21.8
		keylog_0	mlog_2	ot_s_cr_2	ot_s_rt_2				
Physical stress	1.23	0.273	−11.4	−0.137	−0.504	−0.0228	0.826	0.874	17.5
		sleep_0	mlog_1	ot_s_r_1	ot_s_rl_1	ot_s_cr_2			
Total score	−4.79	−0.135	1.28	14.3	0.0116	0.252	0.762	5.22	17.4
		sleep_0	ot_v_r_0	ot_s_cr_1	ot_v_rt_1	ot_s_l_2			
K6	8.14	−0.007028	0.339	−27.5	49.6		0.738	2.88	12.0
		ot_v_rt_1	sleep_2	keylog_2	mlog_2				

According to Table 3, the correlation coefficients were 0.6 for depression and anxiety and 0.7–0.8 for others. Each error ε for each mental score, lassitude, irritation, fatigue, anxiety, depression, or physical stress was about 1, which is almost the same as the minimum scale of score. The errors for total score and K6 were 5.2 and 2.9, and the full scores for total score and K6 were 30 and 24, respectively. Each error was within 24% of each full score. Considering the accuracy of the subjective score sheet, these errors seem acceptable for practical use.

**Figure 4:**
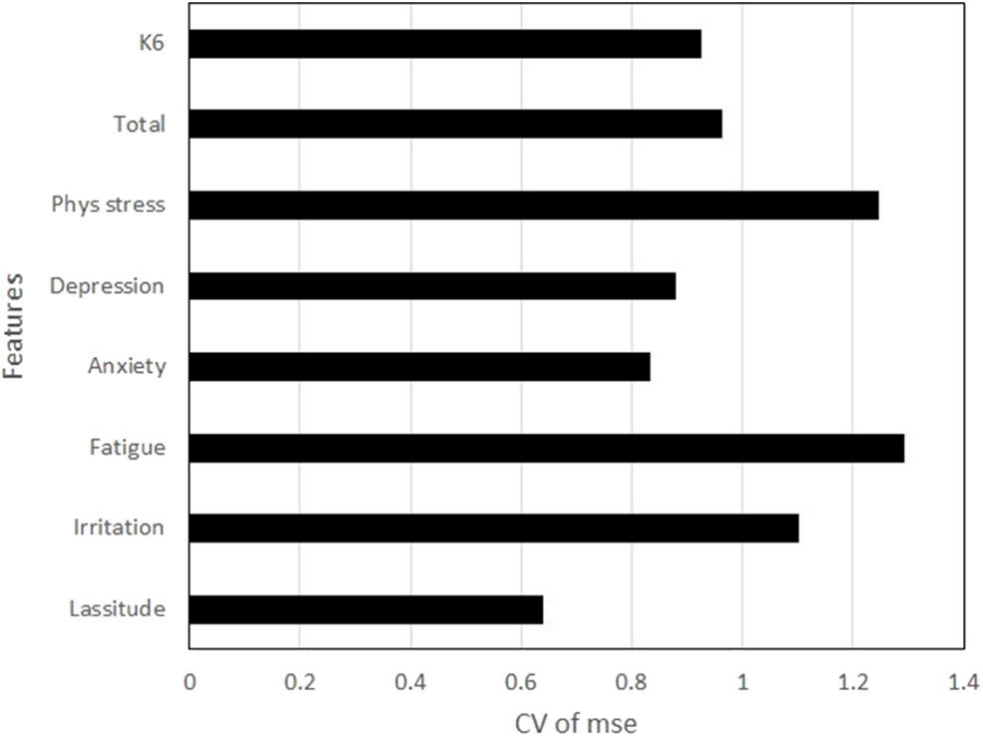
Coefficients of variation of mean squared errors (mse) across participants for the target variables.

The coefficients of variation (CVs) of mean squared errors across participants for the target variables were around one (Figure 4). When CV is one, the standard deviation is equal to the mean value. The effect of participant dependency on models was remarkable, although it had been tried to be controlled through the participant-wise cross validation. Other participant features, such as gender and age, were not controlled in this study due to the small number of data. Case analysis potentially improves the robustness of model. Increasing the number of participants data possibly provide the models with much smaller participant dependency.

We should point out here that the optimum model for K6 consisted of explanatory variables measured one or two weeks before the mental score. As such, these multimodality measurements predicted the mental condition before participants made a subjective complaint. Because K6 is used for screening mental distress, the system shows potential as an early screening technique.

Major participants were healthy volunteers. Neither clinical diagnosis nor intervention were performed according to the results obtained in this study. Therefore, the effectiveness of this system for screening and preventing mental distress has not been confirmed yet. This system remains to be investigated further. Even though the current results were preliminary, this system showed a promising function to replace the conventional mental health care services based on manual questionnaire sheets.

## Conclusion

We developed a monitoring system for mental condition involving the PC logger, the activity tracker, and Optical Topography (OT). We collected the biometric data from thirty-nine healthy volunteers in office for more than four months. The multivariate linear models for mental scores of BJS and K6 were obtained by using the supervised machine learning and the cross-validation. Those models included several variables from the collected biometric data such as the fractal dimensions of PC operation obtained from the PC log, steps, METs, and sleeping time from the activity tracking log, brain activities, laterality, correction rates and response time during working memory tasks from the brain activity and performance log. Each mental score estimated by each model was well agreed with each score of questionnaire. Especially, K6 score was estimated by using the biometric data collected from one or two weeks before. The system is potentially useful for the mental healthcare including the prevention.

## Data Availability Statement

The datasets generated for this study will not be made publicly available. It was not included in the ethics approval to submit the datasets.

## Ethics Statement

The studies involving human participants were reviewed and approved by the internal review board of Central Research Laboratory, Hitachi, Ltd. The patients/participants provided their written informed consent to participate in this study.

## Author Contributions

MK conceived the concept and led the project. SS and MK analyzed the data. HA, AN, and AO performed the field trial and the data preprocessing. HN developed the platform and database. ME and HK developed the PC logger and the activity tracker and analyzed each data, respectively. MK, HA, and TF developed the wearable OT. All authors contributed to the article and approved the submitted version.

## Conflict of Interest

All authors were employed by company Hitachi, Ltd.

## Footnotes

^1^*Web Page of International Employee Assistance Professionals Association (EAPA)*. Available online at: https://www.eapassn.org/FAQs

^2^*The Brief Job Stress Questionnaire English version*. Available online at: https://www.mhlw.go.jp/bunya/roudoukijun/anzeneisei12/dl/160621-1.pdf

^3^Available online at: http://www.tmu-ph.ac/topics/pdf/sotenkansan.pdf (in Japanese).
